# Deep Learned Segmentations of Inflammation for Novel ⁹⁹^m^Tc-maraciclatide Imaging of Rheumatoid Arthritis

**DOI:** 10.3390/diagnostics13213298

**Published:** 2023-10-24

**Authors:** Robert Cobb, Gary J. R. Cook, Andrew J. Reader

**Affiliations:** 1 Department of Biomedical Engineering, School of Biomedical Engineering and Imaging Sciences, King’s College London, London WC2R 2LS, UK; andrew.reader@kcl.ac.uk; 2 Department of Cancer Imaging, School of Biomedical Engineering and Imaging Sciences, King’s College London, London WC2R 2LS, UK; gary.cook@kcl.ac.uk; 3 King’s College London and Guy’s and St Thomas’ PET Centre, King’s College London, London WC2R 2LS, UK

**Keywords:** ⁹⁹^m^Tc-maraciclatide imaging, deep learning, AI, rheumatoid arthritis

## Abstract

Rheumatoid arthritis (RA) is an autoimmune disease that causes joint pain, stiffness, and erosion. Power Doppler ultrasound and MRI are imaging modalities used in detecting and monitoring the disease, but they have limitations. ⁹⁹^m^Tc-maraciclatide gamma camera imaging is a novel technique that can detect joint inflammation at all sites in a single examination and has been shown to correlate with power Doppler ultrasound. In this work, we investigate if machine learning models can be used to automatically segment regions of normal, low, and highly inflamed tissue from 192 ⁹⁹^m^Tc-maraciclatide scans of the hands and wrists from 48 patients. Two models were trained: a thresholding model that learns lower and upper threshold values and a neural-network-based nnU-Net model that uses a convolutional neural network (CNN). The nnU-Net model showed 0.94 ± 0.01, 0.51 ± 0.14, and 0.76 ± 0.16 modified Dice scores for segmenting the normal, low, and highly inflamed tissue, respectively, when compared to clinical segmented labels. This outperforms the thresholding model, which achieved modified Dice scores of 0.92 ± 0.01, 0.14 ± 0.07, and 0.35 ± 0.21, respectively. This is an important first step in developing artificial intelligence (AI) tools to assist clinicians’ workflow in the use of this new radiopharmaceutical.

## 1. Introduction

Currently, ultrasound and MRI are widely used to detect and monitor inflamed tissue in rheumatoid arthritis (RA). ^99m^Tc-maraciclatide imaging has been shown to correlate well with power Doppler ultrasound [1] and is being investigated as an adjunct to these imaging modalities. Unlike MRI, ^99m^Tc-maraciclatide is associated with ionizing radiation but is widely available and faster than MRI and can scan all joints in a single scan, unlike ultrasound, where each joint is scanned individually. These other modalities have been used to monitor RA for some time, and there are studies on the use of artificial intelligence (AI) models to help with early detection [2] and monitoring of the disease [3]. This includes early grading of RA using MRI [4] and classifying metacarpophalangeal joints using ultrasound images [5].

AI tools are increasingly being incorporated into clinicians’ workflows to help with clinical efficiency [6] and improve interobserver agreement [7]. Image segmentation is one such task that has been studied extensively [8,9,10], with various methods and tools, such as data augmentation with generative adversarial networks [11,12], cascade networks [13], and deep supervision [14], that can be used to improve performance.

In terms of AI imaging diagnostics in RA, there have been several recent papers on the topic, such as [15] where Alarcón-Paredes et al. used thermal and RGB images as well as other collected features (weight, height, age, etc.) from female patients to determine if the patient has RA. There are also reports [16,17] in which RA diagnosis is determined from a convolutional neural network (CNN) based on hand X-ray images. In [18], similar to [15], RGB images were used with questionnaire information to diagnose RA.

**Figure 1:**
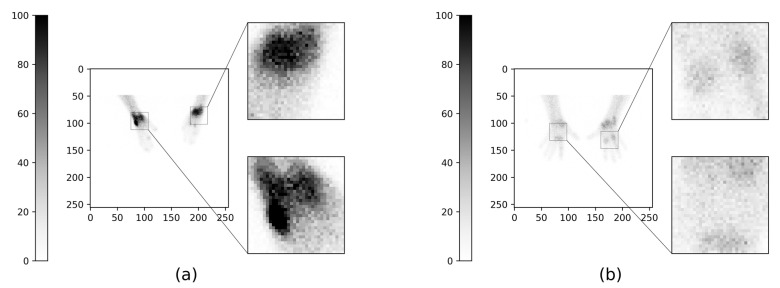
2 Example planar ⁹⁹^m^Tc-maraciclatide scans of the hands showing focal joint inflammation with some inflamed regions magnified. (**a**) shows an oblique view, and (**b**) shows a palmar view.

**Figure 2:**
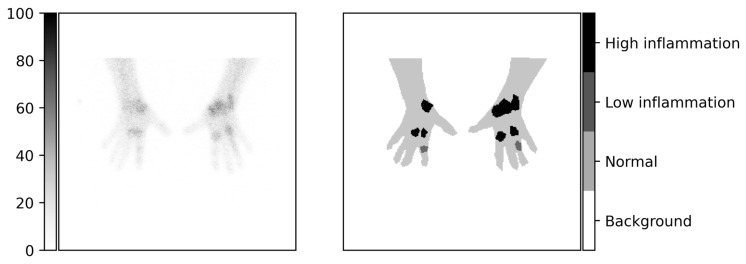
Example scans with clinically segmented labels of normal, low, and high inflammation of the tissue. Labels were segmented nonexclusively (each pixel can be labeled multiple times), and the label with the highest degree of inflammation was taken per pixel to force exclusivity. More information on this can be found in Section 2.1.

In this study, our aim is to build AI tools for ^99m^Tc-maraciclatide imaging to detect inflamed tissue in RA. Our hypothesis is that machine learning models can be used to automatically segment normal and inflamed tissue of patients’ hands (Figure 1 and Figure 2) with RA.

## 2. Materials and Methods

**Figure 3:**
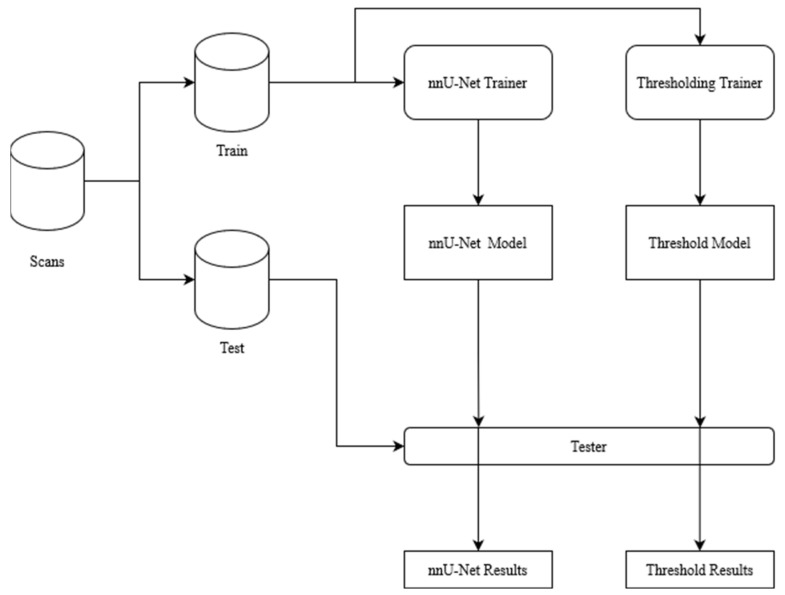
Overview of training and testing of each model (threshold and nnU-Net described later in Section 2.2 and Section 2.3, respectively) for a single crossfold of data. The scans were separated into 90% train and 10% test; the two models were trained on the 90%. Once trained, the models were tested on the held-out 10% test dataset.

A thresholding and nnU-Net model were trained and compared. The overview of the methodology for a single crossfold can be seen in Figure 3.

### 2.1. Data

The data were collected as part of a previous study and consisted of 192 hand and wrist images (images were of the size 256 × 256) from 48 patients, including palmar, dorsal, and two oblique views (Figure 1). Each patient had an injection of 752 ± 32 MBq of ^99m^Tc-maraciclatide, and after a two-hour period, the hand views (as well as feet and whole-body views) were acquired using a gamma camera over a 1 h scanning period. The images were then manually segmented into non-mutually exclusive regions of normal, low, and high inflammation by a clinician (GC) experienced with ^99m^Tc-maraciclatide imaging in RA. To make the labels mutually exclusive, i.e., one classification per pixel, the more severe class was taken. For example, a pixel that was classified as both high and low inflammation was marked as only highly inflamed, and pixels that were classified as normal and low inflammation were marked as low inflammation. Any pixels that were not classified as normal, low, or high inflammation were marked as background. The images were segmented using label-studio [19], where the images were exported with fixed color scaling from 1 to 100 based on the clinician’s decision after being shown a selection of images for different color scales (Figure 2).

### 2.2. Thresholding

**Figure 4:**
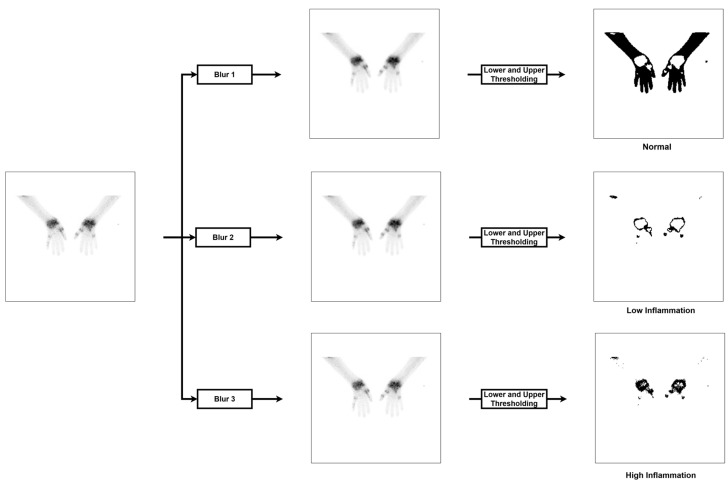
Thresholding model. The input image is first blurred by a single Gaussian kernel and then segmented using a lower and upper threshold for each of the three possible labels, resulting in 9 trainable parameters for the model. Each label is learned independently, and therefore, a pixel can be detected as multiple classes.

To serve as a baseline model, we trained a simple thresholding model to segment regions of normal, low, and highly inflamed tissue. The thresholding model consisted of first smoothing the input image using a Gaussian kernel followed by lower and upper threshold values of the smoothed image (Figure 4). The width of the Gaussian kernel is characterized by the sigma value (σ), and the neighborhood size of the kernel in terms of pixels is based on Equation (1).

(1) $$kernel\_size=\left\{\begin{array}{c} round(\max\left(3\sigma ,\;1\right))+1\;if\;round(\max\left(3\sigma ,\;1\right))\;is\;even\;\\ \;\;round\left(\max\left(3\sigma ,\;1\right)\right)\;\;\;\;otherwise\;\;\; \end{array}\right.$$

Each of the three labels, normal, low, and high inflammation, was learned independently, leading to a model that consists of only nine trainable parameters (a sigma value, lower and upper threshold values for each of the three labels).

The sigma, lower, and upper threshold values were selected by grid search over the training dataset. The search values of σ were from 0.1 to 4.0 every 0.1, and for the lower and upper threshold values, the search was in the range from 0 to the maximum value in the training dataset. The dice score was used to select the model parameters that performed the best over the validation dataset. The thresholding parameters that performed the best over the validation dataset in the inner crossfold were then tested on the test dataset; more information on the crossfolding can be found in Section 2.8.

### 2.3. nnU-Net

We also used the nnU-Net [20] model to segment the regions of inflammation in the images. The nnU-Net model is a convolutional neural network biomedical imaging segmentation framework. It uses a U-Net [21] style convolutional neural network with instance normalization and leakyReLU activation functions. It uses a stochastic gradient descent (SGD) optimizer with a combined Dice and cross-entropy loss trains for 1000 epochs with a polynomial learning rate scheduler (a scheduler that decreases the learning rate in accordance with a polynomial function). The nnU-Net model uses data augmentation strategies such as rotations and adding Gaussian noise. The nnU-Net framework trains three models: a 2D U-Net, a 3D U-Net, and a (3D) cascade U-Net; however, due to our data only being 2D, only the 2D U-Net model is trained and evaluated. The nnU-Net model version used was the version 1 model, and the code can be found in the GitHub repository: https://github.com/MIC-DKFZ/nnUNet/tree/nnunetv1 (accessed on 13 March 2023); the trained weights are available on request.

### 2.4. Intraobserver Variability

In addition to the two models being compared, the clinician who segmented the data also re-segmented ≈15% of the data a month after the initial data were segmented using the same labeling procedure outlined in Section 2.1. The re-segmented images were compared to the original segmentations using the same metrics to give a comparison of how the AI models compare to a human observer. Standard deviation numbers are based on crossfolds and are therefore not available for the intraobserver data.

### 2.5. Dice Score

A modified Dice score was used for the evaluation of the models. The regular Dice score is defined in Equation (2). Due to the dataset having a large number of empty segmentation maps (56 images have no low inflammation, 87 images have no high inflammation, and 45 images have neither low nor high inflammation) and the known issues that the Dice score has with empty segmentations as discussed in [22], we used a modified version of the Dice score (Equation (3)) to define the comparison of two empty segmentation maps to give a modified Dice score of 1 as opposed to undefined in the initial formulation. The Dice score from the MONAI [23] data package was used for the modified Dice metric.

(2) $$Dice\;Score=\frac{2\left|X\cap Y\right|}{\left|X\right|+\left|Y\right|}$$

(3) $$Modified\;Dice\;Score=\left\{\begin{array}{c} \frac{2\left|X\cap Y\right|}{\left|X\right|+\left|Y\right|}\;if\;\left|X\right|+\left|Y\right|\;\neq 0\\ \;1\;\;\;otherwise \end{array}\right.$$

### 2.6. Intersection over Union (IoU)

A modified IoU score was also used for the evaluation of the models. Similar to the modified dice score above, the regular IoU (Equation (4)) score is modified to account for empty segmentation comparison (Equation (5)). The IoU metric from the MONAI [23] data package was used for the modified IoU metric.

(4) $$IoU\;Score=\frac{\left|X\cap Y\right|}{\;\left|X\cup Y\right|}$$

(5) $$Modified\;IoU\;Score=\left\{\begin{array}{c} \frac{\left|X\cap Y\right|}{\;\left|X\cup Y\right|}\;if\;\left|X\cup \;Y\right|\;\neq 0\\ \;1\;\;\;otherwise \end{array}\right.$$

### 2.7. Confusion Matrix

To further analyze the results of the model, confusion matrices were used to show the breakdown of the proportion of pixels classified correctly and incorrectly, including what misclassified pixels were classified as. These matrices were obtained for both trained models as well as for analyzing the intraobserver variability. The model confusion matrices were calculated over all outer crossfolds, and the intraobserver confusion matrix was taken over the ~15% of data that was re-segmented.

### 2.8. Crossfolding

**Figure 5:**
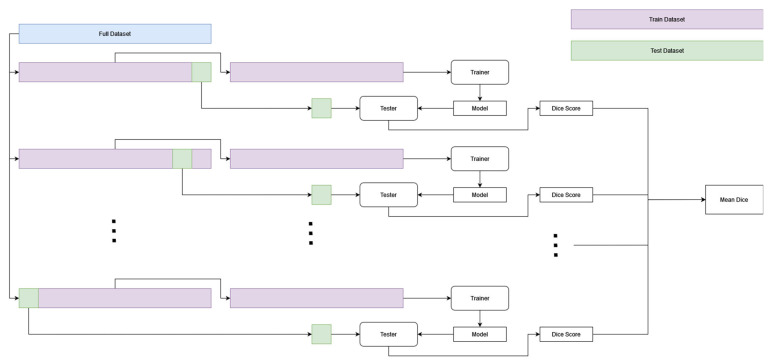
Cross-folding diagram. Each model is trained and evaluated 10 times by using a different separate 10% of the dataset to evaluate the model and the other 90% to train the model.

To limit bias in the assessment of our models, we used crossfolding to create 10 models for the nnU-Net model as well as for the thresholding model. The dataset was partitioned into 10 buckets, each bucket consisting of 4 or 5 patients so that no patient images were shared across a bucket. One bucket was reserved as a held-out test dataset. The other nine buckets were used as a train/validation dataset (Figure 5). Each image within the overall dataset exists in the test dataset for one crossfold and in the train/validation dataset for nine crossfolds. Results presented show means ± standard deviations based on these crossfolds. A box-whisker plot is shown using the variance in results presented using different crossfolds.

For the thresholding model, an inner crossfold was also used. For each outer crossfold, we trained nine models, one for each of the nine train/validation buckets being held as a validation dataset. The thresholding model was trained on eight buckets of data, and the model that performed best on the validation bucket was then tested using the held-out test dataset so that 10 models in total were tested on the test dataset.

### 2.9. Single Inflammation Class

**Figure 6:**
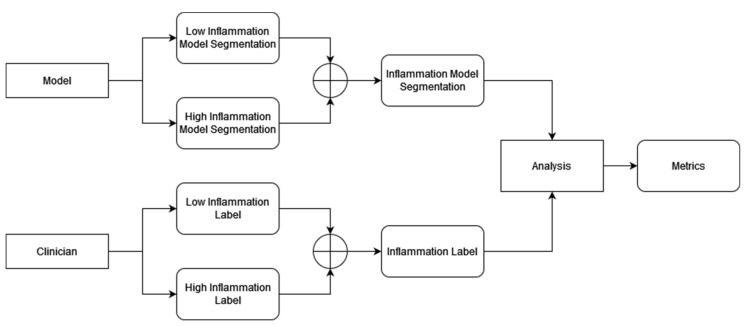
Low and high inflammation classes can be combined after model segmentation to create a single inflammation class. This can then be compared to the combined inflammation class created by combining the low and high classes segmented by the clinician. The combination is performed using pixel-wise addition of the segmentation maps. Thresholding model segmentations are clipped.

The model segmentations and the labels split the inflammation into two classes: low and high inflammation. The two classes can be combined into one “inflamed” class by adding the labels together. The mutually exclusive constraint introduced prior ensures that the addition of the labels results in a new valid label, as no pixel is marked as both low and high inflammation. The models can then be compared with respect to this combined class. The models still make two separate segmentations for both low and high inflammation, but the two classes are then combined and analyzed. Note that this is different from training two additional models that are trained to output a single inflamed class. (Figure 6).

### 2.10. ROC Curves

We also retroactively analyzed our generated segmentation maps as a classification task by classifying an image as inflamed if the corresponding label contains at least one pixel of inflammation (high or low). Using this, we created a ROC curve using the number of pixels classified as inflammation in the prediction as our variable threshold. For example, when 0 inflamed pixels are needed to classify an image as inflamed, we gain a true positive rate and false positive rate of 1; when the number of pixels needed to classify the image as inflamed is equal to the entire number of pixels in the image, we get a true positive rate and false positive rate of 0.

## 3. Results

**Figure 7:**
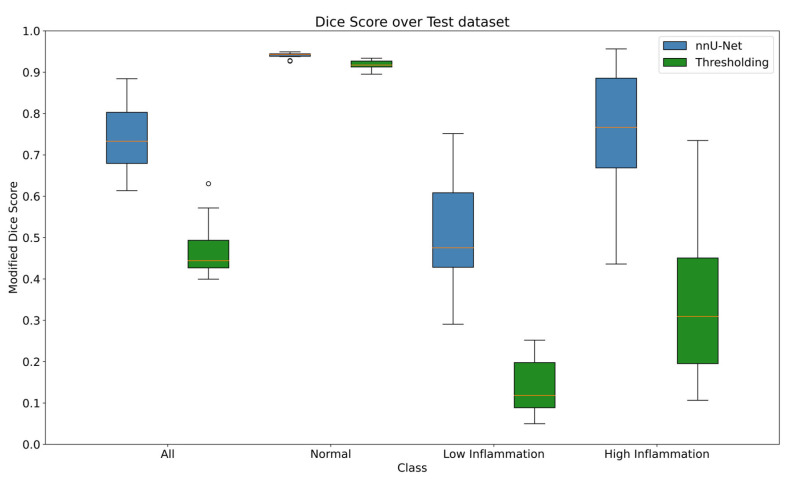
Modified Dice score performance of the thresholding and nnU-Net models for the three classes as well as for all classes combined. The box-whisker plots represent the distribution in modified Dice score values based on the 10 models obtained from the 10 crossfolds. The boxes span from the lower to upper quartiles, showing the median in red. The whiskers extend to a maximum of 1.5 times the interquartile range but end at the lower/largest data point within the range; outliers beyond this range are plotted as circles.

Box-Wisker plots of the nnU-Net and thresholding models are shown in Figure 7. The nnU-Net model segmented inflammation with modified Dice scores of 0.94 ± 0.01, 0.51 ± 0.14, and 0.76 ± 0.16 for normal, low inflamed and highly inflamed tissue. The thresholding model, in contrast, gave modified Dice scores of 0.92 ± 0.01, 0.14 ± 0.07, and 0.35 ± 0.21 (Table 1). The results of the nnU-Net model are comparable to the results of the intraobserver-modified Dice scores of 0.94, 0.51, and 0.63.

We also computed modified IoU scores for the nnU-Net as 0.89 ± 0.01, 0.43 ± 0.15, and 0.70 ± 0.15 for the normal, low, and high channels, respectively. For the thresholding model, the modified IoU results were 0.85 ± 0.02, 0.11 ± 0.07, and 0.28 ± 0.18 for normal, low, and high inflammation, respectively.

**Figure 8:**
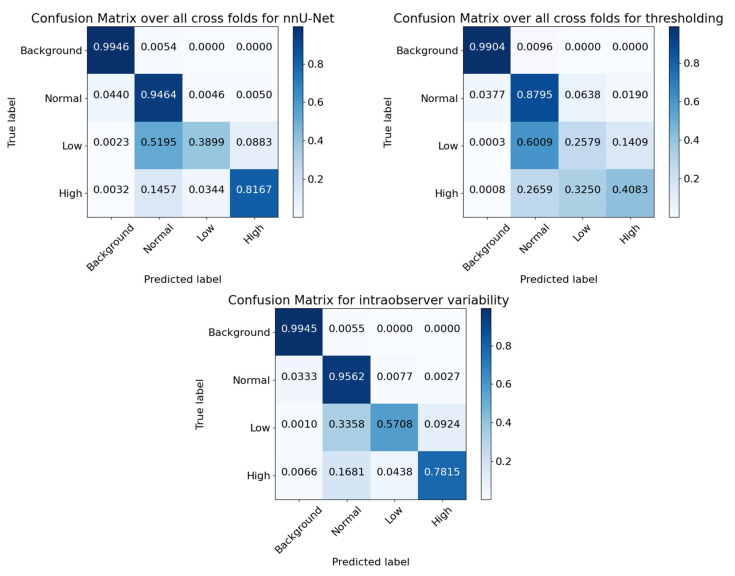
Normalized pixel confusion matrices for the two models and the human intraobserver measurements. Confusion matrices were plotted with modified code from http://scikit-learn.org/stable/auto_examples/model_selection/plot_confusion_matrix.html (accessed on 28 August 2023).

The confusion matrices (Figure 8) show that the nnU-Net model accurately classifies over 99% of background pixels, over 94% of normal tissue pixels, and approximately 39% of low and 82% of highly inflamed tissue pixels. For the low inflammation class, where the model performance is weakest, the model predicts the low inflammation tissue as normal tissue ~52% of the time and predicts it as highly inflamed ~9% of the time. Interestingly, when the model inaccurately classifies highly inflamed tissue, it does so as normal tissue (~15%) more than it does low inflamed (~3%). The thresholding confusion matrix, in comparison, shows less accuracy for all classes. The thresholding model misclassifies high inflammation, mostly as low inflammation, as one would expect, as opposed to the nnU-Net model, which mostly misclassifies high inflammation as normal tissue. Lastly, a confusion matrix for the intraobserver variability is presented. This shows that the intraobserver variance is relatively large for regions of low and high inflammation.

**Figure 9:**
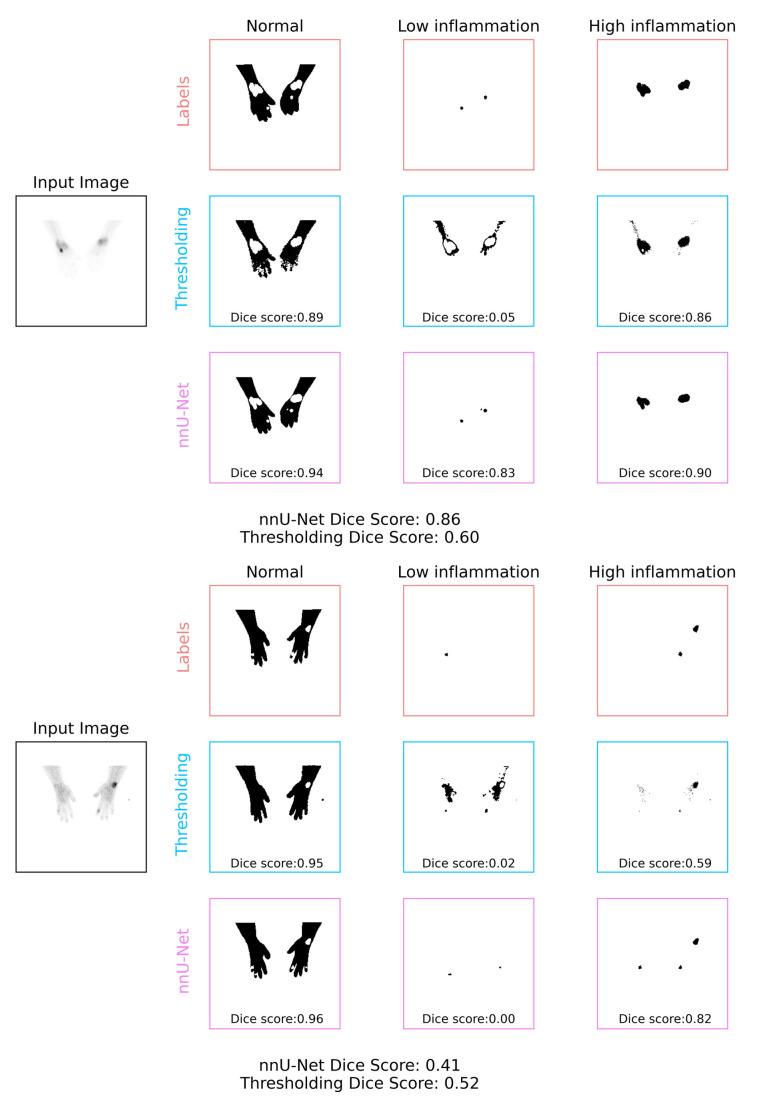
Two example sets of generated labels for Thresholding and nnU-Net models.

Looking at some example segmentations, we can see the thresholding model predicting several areas of small amounts of inflammation all over the hand image as the decision made using the thresholding is relatively local. The nnU-Net model, in contrast, shows a smaller number of large areas of inflammation being predicted, more in line with the labels. The examples also show that the nnU-Net model is penalized in the modified Dice score for errors in boundary prediction (Figure 9).

### 3.1. Inflammation as a Single Class

In addition to looking at high and low inflammation separately, we also evaluated the model performance when combining these two classes into one “inflammation” class. If the two classes predicted by the nnU-Net model are combined into a single inflamed class and compared to the combined inflammation in the clinically segmented labels, we get a modified Dice score of 0.72 ± 0.12. This, again, is broadly in line with the intraobserver modified Dice score of 0.76 when collapsing the inflammation in the same manner. The score of the inflamed channel for the nnU-Net model (0.72 ± 0.12) is much closer to the model’s high-inflammation-modified Dice score of 0.76 ± 0.16 than the low-modified Dice score of 0.51 ± 0.14. This is in part due to the fact that mistakes in inflammation classification are not counted against the model in this way of analyzing the data (i.e., errors of misclassifying low inflammation as high inflammation and vice versa are ignored when just considering inflammation as a single class). This could also be due to the relative sizes of high inflammation and low inflammation in the data; approximately 0.42% of all the pixels are classified as highly inflamed compared to 0.25% as low inflammation.

The thresholding model, in comparison, gave results of 0.40 ± 0.18 when analyzed in this manner. This significantly underperformed in comparison to the nnU-Net model and shows again that the combined inflammation is much closer to the higher modified Dice score of 0.35 ± 0.21 than 0.14 ± 0.07 given by the high inflammation than the low inflammation. Table 2 shows the results of combining the class for each crossfold for the two models.

### 3.2. ROC Curve

**Figure 10:**
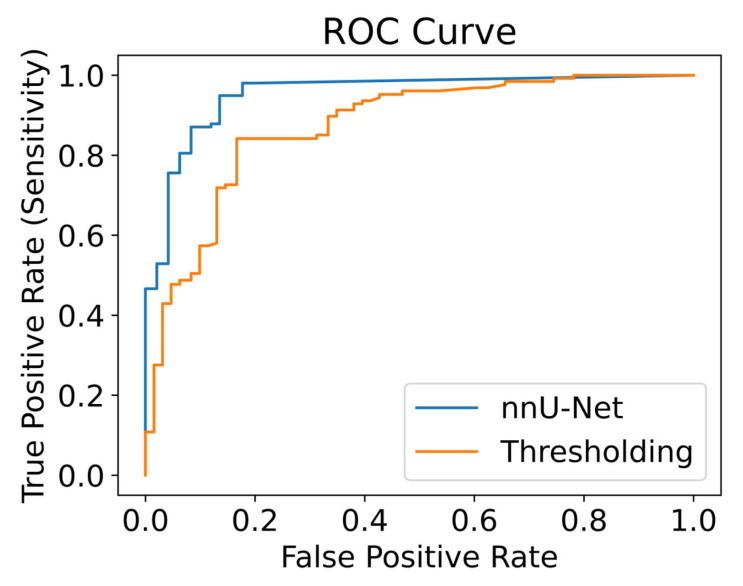
ROC curve calculated by varying the threshold number of pixels in the prediction to classify the image as inflamed.

The ROC curve is shown in Figure 10, and the calculated area under the curve (AUC) is 0.96 for the nnU-Net model and 0.88 for the thresholding model. For the case of using 1 pixel in the prediction as our threshold, we gain a sensitivity of 0.98 ± 0.06 and specificity of 0.80 ± 0.31 for the nnU-Net model and a sensitivity of 1.00 ± 0.00 and specificity of 0.22 ± 0.36 for the thresholding model.

## 4. Discussion

This study is the first to investigate automatic segmentation of inflammation in RA patients with ^99m^Tc-maraciclatide; as such, direct comparisons are hard to find in the literature. There are studies in the literature that have automatically tried to classify arthritic disease activity via Color Doppler ultrasound [24] and segment regions of inflamed tissue with MRI [25]. In [24], the authors report an accuracy rate of 75.0% when predicting an OMERACT-EULAR Synovitis Scoring (OESS) score over the entire US image and 87% when predicting healthy vs. diseased. For the healthy/disease model, they also report sensitivity and specificity of 0.864 and 0.875. Comparing these numbers to our calculated sensitivity and specificity for the nnU-Net model shows we have a higher sensitivity rate (0.98 vs. 0.864) but lower specificity (0.80 vs. 0.875). They also use a larger dataset of 1342 images, which can greatly affect the performance of machine learning models. The research presented here differs in that we are segmenting instead of classifying and using a completely different modality.

### 4.1. Relative Performance of Low and High Inflammation

Both models and the intraobserver comparison showed better performance at segmenting high inflammation than low inflammation. When looking at the confusion matrices, it seems that low inflammation tissue is confused for normal tissue more than it is high inflammation. This could partly be because of the lack of examples of low inflammation in the training dataset relative to high inflammation, as mentioned previously (0.42% of pixels for high and 0.25% of pixels for low). However, in addition to this pixel level imbalance, if we analyze the average amount of pixels in an ‘inflamed’ lesion, we get 62.81 ± 82.55 pixels for low and 121.25 ± 130.65 pixels for high inflammation lesions. This shows that not only are there fewer pixels of low inflammation to learn from but that, on average, the high inflammation is split into a larger number of smaller lesions. This could also contribute to the low intraobserver modified Dice score, as boundary imperfections will have a larger impact than drawing larger, fewer lesions.

### 4.2. Imbalanced Dataset

There have been several attempts in the literature to create loss functions that help with training on imbalanced datasets, such as focal loss [26] that modifies cross-entropy loss to help handle class imbalance and Tversky loss that has been shown to outperform Dice loss in some tasks [27].

The nnU-Net model uses traditional augmentation, such as Gaussian blurring and rotations, to boost the train dataset size. There has been widespread adoption of generative augmentation techniques such as generative adversarial networks (GANs) [28] and, more recently, diffusion models [29] that could be used to augment the dataset with synthetic samples. Papers using synthetic generation have, in some cases, shown significantly improved results [30,31,32] and could be used here to improve the results of our model, especially with the low, high inflammation imbalance similar to [33].

### 4.3. Limitations

The research presented here currently only predicts clinically segmented labels, which, in this instance, have demonstrably high variability at the pixel level. The work is also based on the Dice score, which, whilst a standard metric in machine learning and medical imaging, has known issues with empty segmentations; it is also questionable if such a metric is suited to our task as the exact boundaries of the inflamed tissue are less clinically relevant than simply predicting if a specific joint has any inflammation. In addition, our labels are based on a single observer, and a better understanding of the data may arise from looking at predictions from multiple observers. Future work is needed to investigate these issues.

The work presented here is also limited in that the dataset is relatively small and is collected from only one center and thus may not be representative of data collected from other geographical sites/regions. Further work is warranted to study the generalisability of the models presented here.

## 5. Conclusions

In conclusion, two machine learning models were trained and compared on the task of segmenting normal tissue and regions of inflammation in the hands of patients with RA using ^99m^Tc-maraciclatide imaging. The nnU-Net model shows a promising ability to segment regions of low and highly inflamed tissue, with similar performance to a human observer in terms of repeatability for segmenting normal and highly inflamed tissue. The nnU-Net model also outperformed a thresholding model built for this task. Further work may include getting multiple trained observers to segment the images and study the interobserver variance, as well as using these multiple labels to learn a more robust model and quantify uncertainty. We believe this work is the first step in building a fast and reliable clinical assistance pipeline to use this new radiopharmaceutical in conjunction with other modalities to improve efficiency in detecting and monitoring synovitis in RA patients.

## Figures and Tables

**Table 1:** Modified Dice score segmentation results by crossfolding over the held-out test dataset. Best highlighted in bold.

Crossfold	Thresholding Model				nnU-Net Model			
	Normal	Low	High	All	Normal	Low	High	All
1	0.93	0.2	0.28	0.47	0.95	0.61	0.72	0.76
2	0.93	0.08	0.49	0.5	0.94	0.38	0.8	0.71
3	0.91	0.12	0.32	0.45	0.94	0.43	0.65	0.67
4	0.91	0.19	0.11	0.4	0.95	0.46	0.44	0.61
5	0.92	0.05	0.33	0.43	0.94	0.64	0.93	0.83
6	0.92	0.11	0.68	0.57	0.94	0.5	0.84	0.76
7	0.91	0.24	0.73	0.63	0.94	0.61	0.9	0.82
8	0.92	0.25	0.13	0.44	0.93	0.29	0.64	0.62
9	0.93	0.11	0.17	0.4	0.94	0.75	0.96	0.88
10	0.90	0.08	0.3	0.43	0.93	0.43	0.73	0.7
Mean	0.92 ± 0.01	0.14 ± 0.07	0.35 ± 0.21	0.47 ± 0.08	**0.94 ± 0.01**	**0.51 ± 0.14**	**0.76 ± 0.16**	**0.74 ± 0.09**

**Table 2:** Modified Dice score segmentation results by crossfolding over the held-out test dataset when collapsing low and high inflammation into a single class. Best highlighted in bold.

Crossfold	Thresholding Model	nnU-Net Model
1	0.34	0.73
2	0.5	0.68
3	0.39	0.67
4	0.2	0.66
5	0.26	0.83
6	0.68	0.82
7	0.72	0.83
8	0.28	0.5
9	0.21	0.89
10	0.42	0.63
Mean	0.4 ± 0.18	**0.72 ± 0.12**

## Data Availability

Data is provided by Guy’s and St Thomas’s NHS Trust and, due to privacy and ethical reasons, cannot be shared at this time.
